# A monetary model of global peace and health

**DOI:** 10.1186/s12992-024-01029-9

**Published:** 2024-04-09

**Authors:** Iman Bastanifar

**Affiliations:** Department of Economics, Faculty of Administrative Sciences and Economics, University of Isfahan, Isfahan, Iran

**Keywords:** Global Peace, COVID-19, Money, Shopping time, E58, N40

## Abstract

**Background:**

This study aims to expand on the concept of peace and health by drawing from Keynes' theory of the economic consequences of peace, in light of the global pandemic experienced in 2020 due to COVID_19.

**Methods:**

In this paper, I will elaborate on the concept of ‘security’, as an indicator of peace in the time of biological shocks, in order to expand the definition of Keynesian precautionary motivation. This puts forth a new monetary policy model developed to make contributions to achieving global peace. In so doing, I will calculate the optimal growth rate of discount rate through utilizing the Global Peace Index (GPI), adjusted by the Case Fatality Risk (CFR) of COVID-19 in a dynamic shopping time monetary model. This analysis is comprised of the top 15 GDP countries as well as the 10 most and least peaceful countries in 2020.

**Results:**

The results indicate that households in more peaceful and healthy countries tend to hold less money compared to those in less peaceful and healthy countries. Besides, the discount rate needs to be reduced due to the outbreak of COVID-19 and the decrease in the level of peace in the economy.

**Conclusion:**

Insofar as the imposition of fines through international legal circles on countries with an insignificant health and peace policy will increase the cost of liquidity, other alternative methods of financing will be affor dable for the countries.

## Introduction

Keynes criticized the financial impositions on Germany by the Treaty of Versailles. In his work *The Economic Consequences of the Peace*, Keynes [1] argued that the exertion of financial pressures on German would lead to its economic demolitions as well as that of Europe and the rest of the world. Keynes's expectations became true when the treaty failed, leading to the Locarno Treaties. These treaties enhanced relations between Germany and the other European powers. Therefore, political treaties must consider the economic aspect of peace.

If COVID-19 impacts countries as a global enemy and disrupts international security, peace, and economic stability, a decision must be made to compensate for the resulting losses. This decision-making process in an uncertain situation within the framework of monetary policy should involve determining the underlying variables in monetary economics, such as the discount rate. As Keynes argued in 1920, Insofar as imposing excessive penalties on Germany would be negatively consequential to the economy of the world, opting for a non-optimal discount rate can also lead to negative consequences like inflation. According to Keynes, inflation is the best way to overturn a society based on a capitalist system [2], 79). Therefore drawing on Keynes's idea of "The Economic Consequences of the Peace", a model can be designed to determine the optimal discount rate in the context of COVID-19, therby enhancing economic resilience.

Peace should not be deemed as an out-dated issue which has been solely discussed in economics by Keynes in 1920. This notion, however, has been widely discussed nowadays as one of the Sustainable Development Goals for countries by the United Nations in 2023. According to the United Nations' Sustainable Development report in 2023, progress and modern rough clashes around the world are wrecking the worldwide way to peace [3]. Alarmingly, the year 2022 saw a more than 50 per cent increment in conflict-related civilian passings generally due to the war in Ukraine. At the end of 2022, 108.4 million individuals were coercively uprooted around the globe, with an increment of 19 million compared with the conclusion of 2021 and two and a half times the number of a decadeprior. In 2021, the world experienced the most noteworthy number of deliberate manslaughters within the past two decades. Structural shameful acts, imbalances and developing human rights challenges are putting serene and comprehensive social orders encourage out of reach. To meet the United Nations' Sustainable Development Goals (SDGs) 16 (Peace, Justice, and Strong Institutions) by 2030, taking effective actionsare required to reestablish belief, reinforce the capacity of securing equity for all, and encourage tranquil moves to maintainable advancement.

The concept of peace, within the historical context following the World War I, has largely been examined from myriad of angels in the literature on economics. In this connection, terms such as “defense economics”, “military economics”, “conflict economics”, and “security economics”, have been widely employed to refer to specific aspects of peace, but, more importantly, thus far, the conceptualizations as such have apparently failed to provide an exhaustive explication of how peace-oriented policies at international arenas can lead to peace economics. To add more details to it, peace economics encompasses the designation of political-economic-cultural institutions, and their interrelations and policies are aimed at preventing, mitigating, or resolving any form of latent or actual destructive conflict within and between societies [4], p. 5–6).

**Fig. 1:**
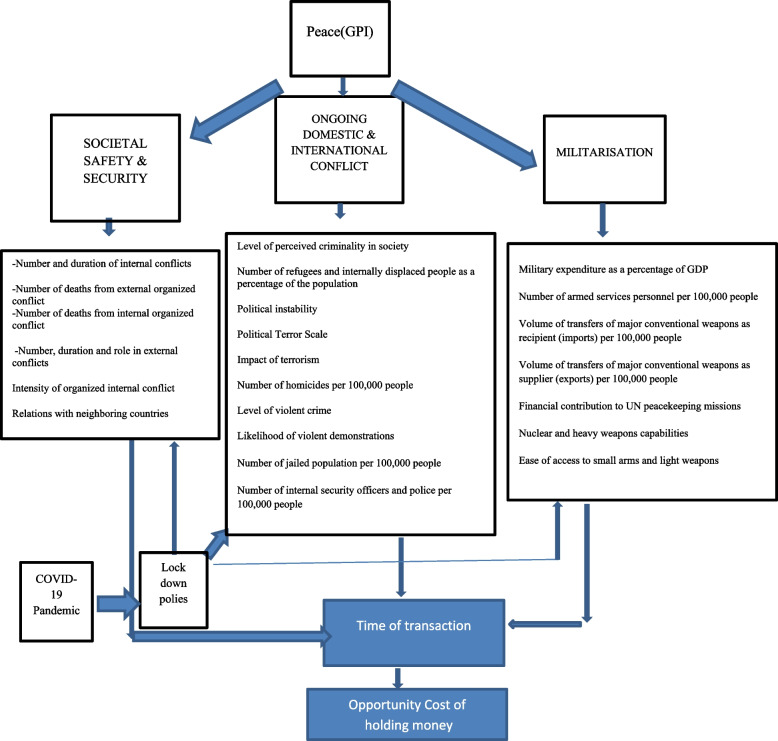
Peace and COVID-19 Pandemic Impacts on the Time of Shopping [Reference author]

The importance of such conflicts in economics stems from the fact that they bring about a sort of uncertainty in economy and escalates the inability of economic agents and policy makers in making precise future forecasts. To mentioning some facts pertaining to the time period between the years 2020 and 2023 can be adantageous. In the advent of COVID-19, in 2020, the monetary policy response was immediate, with a significant expansionary policy. Monetary policy injected liquidity into the system to compensate for the consequences of COVID-19 in some countries such as the US and the Euro area. However, at the end of COVID-19, in 2022, inflation rose and fell in the most countries around the world. This could be attributed to the consequences of COVID-19 and the Russian attack on Ukraine, which led to an increase in consumer prices in 2022 [5]. According to the International Monetary Fund report (data from the World Economic Outlook, April 2023), consumer prices in 2022 increased by more than 8% in the US and Europe. In contrast, China experienced an increase of 1.9%, Japan, 2.5%, Taiwan, 2.9%, Malaysia, 3.4%, Indonesia, 4.2%, and South Korea 5.1. Other emerging countries experienced even higher price increases with India 6.7%, South Africa at 6.9% in, Morocco at 6.6, Egypt at 8.8, Mexico at 7.9 and Brazil at 9.1. For some countries with deeper and longer-term economic crises, Inflation was higher. For example, Turkey experienced a consumer price growth 72.3%, Argentine,72.4%, and Iran 49% in 2022. These economic instabilities are due to a lack of accurate knowledge and quantitative measurable models among economic policymakers, particularly, monetary economists. The risks of the spread of COVID-19 and political tensions worldwide contribute to this situation. Therefore, measuring peace is a way to demonstrate the level of uncertainty in a country and make salient contributions to economic agents and policy makers in making influential economic decisions. Nevertheless, measuring peace is the Achilles heel of the peace economics, especially in the time of globe-wide crises such as the outbreak of COVID-19. Yet, this problem has been partly accounted for by Institute for Economics and Peace (IEP) wherein since 2007, the Global Peace Index (GPI) has been the leading measure of global peacefulness. This index measures 23 indicators across three domains: ‘social safety and security’ ‘ongoing domestic and international conflicts’, and ‘militarization’. Detailed indicators can be shown in Fig. 1.

It is important to note that an increase in the GPI indicates a decrease in the level of peace. Therefore, according to the reports issued by the IEP [6], COVID-19 pandemic has had a negative impact on most of the indicators of the Global Peace Index. Therefore, the level of peace has been influenced by considering the effects of the COVID-19 pandemic (see IEP,  [6]).

The story does not end there. Another challenge *en route* the concept of peace is the discretionary monetary policy making in light of the COVID-19 pandemic. With the outbreak of COVID-19, mounting uncertainty surrounded the economy [7]. As for instance, the implementation of mandatory lockdown policies by health authorities led to the hoarding of essential commodities (e.g., food, medicines) and money [8]. Therefore, to mitigate the economic impact of these lockdown measures, monetary authorities employed extensive debt policies. However, short-run interventions as such, however effective or not, may lack enough resilience in economic terrains. In that, the economic system would be capable of recovering after a shock. Hence, resilience should not be taken as an equivalent for resistance since it pertains to the ability of the system in preventing the occurrence of shocks [4], p.9). In addition to the monetary and economic repercussions, significant social and economic impacts were imposed on households due to the mandatory lockdowns. As a corollary, these policies prevent the realization of stable peace sustainable development in societies. This is because the concept of sustainable development is founded on the belief that there exists a long-term understanding. Therefore, it is in conflict with the nature and function of these policies. These lockdown policies along with short-run monetary policies are akin to injustice and unstable Hidden Treaty Peace or a new Carthaginian Peace, where COVID-19 emerges as the victor and the people of the world are the losers. Hence, it is now imperative for monetary authorities worldwide to devise a monetary rule that incorporates a new peace index accounting for biological shocks or wars. This will help establish a fair and stable global environment for the entire world.

Given all these, in this paper, my aim is to elaborate on the characteristics of an optimal monetary policy by considering the impact of peace on long-run equilibrium. Additionally, I will take into account the existence of the COVID-19 pandemic within a dynamic shopping time model. Furthermore, I will measure the rule for the selected countries.

The structure of the paper is as follows: in the second section, I will provide an overview of the literature on the relationship between uncertainty and money as well as the role of COVID-19 in economic uncertainty. In the third section, I will outline the theoretical-analytical model recruited in this study. In the fourth section, I will calculate the optimal discount rate for the top fifteen GDP countries as well as the ten most and least peaceful countries in 2020 and then present the results. In section five, I will discucss the results, and finally, I will conclude at the end.

## Literature review

### Uncertainty and money relationship

In his book *The General Theory of Employment, Interest and Money*, Keynes [9] believes that one of the reasons for holding money in the liquidity-preference theory is the precautionary-motive. He defines the precautionary motive as "the desire for security as to the future cash equivalent of a certain proportion of total resources".^1^ [9], p.70).

Fielding and Shortland [10] used an econometric model to analyze a period from.

1983–1996 in Egypt. Their findings indicate that financial liberalization and financial stability have resulted in reduction in excess liquidity. However, they also observed that violent political incidents tend to increase in excessive liquidity.

Christopher et al. [11] demonstrated that macroeconomic volatility impacts the cash holding behavior of non–financial firms. They employed the ARCH model and recruited a database spanning from 1957 to 2000. The findings reveal that firms' responses to cash holding behavior were consistent during times of heightened uncertainty. Likewise, Chen et al. [12] have shown that COVID-19 sharply increased the amount of cash in circulation in Canada during March and April 2020. Such a significant increase in demand for cash is triggered by payment systems and precautionary motives for holding money. Thus, it can be argued that variables that affect uncertainty, such as COVID-19, can increase the demand for money by enhancing the precautionary motive to hold money.

### Measuring 2EPC and peaceful index induced by COVID-19

Baker et al. [13] introduce a new index for measuring EPU in the USA. This index utilizes information gathered from newspaper readers. The authors demonstrate that policy uncertainty leads to an increase in divestment and employment reduction in sectors such as defense, health care, finance, and infrastructure construction. Build upon previous research, [14] examines the effect of disasters on economic policy uncertainty. This study measures uncertainty using three indicators, namely: stock market volatility, newspaper-based economic uncertainty, and subjective uncertainty in business expectation surveys. Besides, in a follow-up study, Baker et al., [15], they explore the impact of COVID-19 on economic uncertainty. Their findings reveal that 60 percent of the projected decline in output in the USA can be attributed to the uncertainty of COVID-19.

In the same vein, Al-Thaqeb et al. [16] conducted the research that examined the impact of EPU on the economy. They point out the detrimental effects of governments uncertainty imposes some detrimental effects upon and renders’ decision-making and policies,making it quite difficult for economic authorities. The authors found that high levels of EPU have consequences for all aspects of economic system. They argue that EPU should be taken in to account as a risk management factor in government decision-making. Furthermore, they highlighted that the COVID-19 pandemic has further increased EPU and uncertainty surrounding monetary policy.

In IEP [17], Global Peace Index (GPI), is applied to measure the levels of peacefulness in countries from 2007 to the present. This index consists of three domains such as social safety and security", "ongoing domestic and international conflict" and "militarization". Each domain includes several indicators. The indicators for the "social safety and security" domain include “number and duration of internal conflicts", "number of deaths from external organized conflict", "number of deaths from internal organized conflict", "number, duration and role in external conflicts", "intensity of organized internal conflict", and "relations with neighboring countries."

The second domain is the "ongoing domestic andinternational conflict" whose indicators are comprised of the "level of perceived criminality in society", "number of refugees and internally displaced people as a percentage of the population", "political instability ", "political terror scale", "impact of terrorism", "number of homicides per 100,000 people", "level of violent crime", "likelihood of violent demonstrations", "number of jailed population per 100,000 people", and "number of internal security officers and police per 100,000 people." According to these indicators, "social safety and security" and "ongoing domestic and international conflict" focus on both internal and external conflicts, the effects of conflicts, and the channels and structures that create them.

The third domain is "Militarization." The indicators for this domain include "military expenditure as a percentage of GDP", "number of armed services personnel per 100,000 people," "volume of transfers of major conventional weapons as recipient (imports) per 100,000 people," "volume of transfers of major conventional weapons as a supplier (exports) per 100,000 people", "financial contribution to UN peacekeeping missions", "nuclear and heavy weapons capabilities", and "ease of access to small arms and light weapons.” In this domain, what is considered to be important is the human resources involved in military affairs and related equipment. However, there have been no indicators introduced for biological or cyber warfare or structures associated with these type of warfare.

## Model

In this section, firstly, the conceptual model recruited in this study will be introduced. Secondly, the new shopping time model will be discussed in details.

### The conceptual model

In this section, I will embark on analyzing the effect ofthe GPI introduced on shopping time and the opportunity cost of holding money in both environments. This is will be proceeded with-andwithout considering the the COVID-19 pandemic factors.

#### How does the GPI affect shopping time in the absence of the COVID-19 pandemic?

- Example (1): Imagine a household spends two hours per week shopping at a hypermarket A. If, for some reasons, they are unable to reach the hypermarket A due to some obstacles such as gangsters, the household then decides to go to the hypermarket B. The change in the direction can increase the time spent for shopping. It is also assumed that this household does not make purchases online. If these gang groups are formed as a result of racist or factional policies within a country or if they are influenced by terrorist groups, the disruption of security increases the cost of exchange opportunities. In this example, it means an increase in shopping time.
- Example (2): Imagine a household living in country C which is currently under imposed trade sanctions. Previously, the country C was not sanctioned for receiving goods X and could import the required goods without any trouble from country A. However, the country C has recently been sanctioned and now has to find alternative ways to purchase good X. For instance, they could ask country B as a mediator to buy from country A and deliver it to country C. It goes without saying that this mediation will increase the time for the sanctioned country to purchase the required goods. Besides, other types of sanctions, such as financial sanctions or Swift sanctions can further prolong the trading process. Therefore, foreign conflicts such as sanctions will add to the cost of exchange opportunities, as demonstrated in this example by the increased shopping time.

Examples 1 and 2 demonstrate that the rise of the GPI indicators that mark a dip in peace, leads to prolonged shopping time. Consequently, given the theory of shopping time, the increased shopping time brings about certain constriction on people's leisure time. As an outcome, extra money has to be spent for the ordinary household provisions. This result is consistent with Keynes's precautionary motive to keep money, which increases the demand for money in uncertain conditions.

#### How does GPI affect shopping time during the COVID-19 pandemic?

Mandatory lockdown policies against COVID-19 outbreak, such as social distancing measures and restrictions on internal movements, imposed negative impacts on shopping time. To abide by the social distancing guidelines, households who were unable or unwilling to shop online had to spend much more time waiting in shopping queues. Furthermore, those who rely on public transportation to shop had to wait longer hours due to reduced capacity requirements for social distancing.

Restrictions on internal movement and stay-at-home orders force households to limit or postpone their shopping time; therefor, they need to think of alternative ways to handle these situations. By utilizing new payment methods such as E-commerce, households can remarkably contract the amount of time spent for shopping processes. Besides, some may choose to disregard health awes and hold onto their financial capacity and pay any penalties incurred as a result of exceeding limits or postponing their shopping time.

Lockdown policies have been shown to increase feelings of aggression [18] and also have been linked to a rise in domestic violence [19]. These effects may contribute to an upturn in criminal behavior, thereby giving raise to the GPI. The enforcement of lockdown policies often  requires the involvement of both policy and military forces. [20]. This militarized response to the COVID-19 pandemic could lead to an escalation in military expenses and could potentially impact the Peace Index.

The spread of COVID-19 has prompted internal conflicts in many countries. For example, Farzanegan and Gholipour [21] investigated over 100 countries worldwide during the pandemic years of 2020 and 2021 and found a positive relationship between COVID-19 mortality rates and internal conflict. Countries whose governments had less support in the fight against the virus are more likely to experience internal tensions. There has been concrete evidence in some countries. For example, in the USA, heavily armed protestors gathered at the Michigan State Capitol building in arrange to to voice their opposition to the governor's lockdown order [22]. In South Africa, police forcefully dispersed anti-lockdown protesters outside [23]. In Israel, protests against a second national lockdown led to violent clashes between pro-government and anti-government demonstrators [24]. Therefore, CFR can have an effect on the indicator of "Number and duration of internal conflicts" as shown in Fig. 1.

Therefore, in a country with stricter health policies, trade restrictions are higher, which in turn has a greater impact on peace. Although the impact of COVID-19 has not been specifically categorized as a domin or indicator of peace variable, according to IEP [6], the COVID-19 pandemic has had negative effects on the indicators of GPI. Lastly, the influence of GPI and COVID-19 on shopping time and the opportunity cost of holding money can be depicted in Fig. 1.

These being said, given the recent developments regarding the concept of "peace" in the Keynesian era, it is worth noting that the term "security" in Keynes' definition of precautionary motives should encompass a wide range of dimensions. The conventional definition of the precautionary motive is defined as "the desire for security regarding the future cash equivalent of a certain proportion of total resources" [9], p.70). I will attempt to expend a new definition of the precautionary motive as a desire for peace regarding the future cash equivalent of a certain proportion of total resources.

### The new shopping time model

Modeling money, allows us to determine the proportion of money circulating in the economy. Considering transaction costs, the shopping time model assumes that the less time we spend shopping, the lower the cost we incur [25].

Equation (1) shows the direct effect of money on the utility function and the indirect impact of money on a shopping time utility function.

1 $${\text{u}}({c}_{t}, {{\text{m}}}_{t}, {{\text{l}}}_{t})\equiv {\text{v}}[{c}_{t,} 1-{n}_{t}-g\left({c}_{t} \right(,{m}_{t})]$$

In a shopping time model, money (${{\text{m}}}_{t})$ decreases the shopping time, g(${c}_{t}$,${{\text{m}}}_{t}$) and increases the leisure time (${l}_{t}$). [26]. Leisure is equal to l = 1-n-${n}^{s}$,where n is the time spent in market employment and ${n}^{s}=$ g(${c}_{t}$,${{\text{m}}}_{t}$) is the time spent for shopping. U and V represent direct and indirect utility of the representative household. The shopping time function, g(${c}_{t}$,${{\text{m}}}_{t}$), is an increasing function of consumption and decreasing function to real money balances:${g}_{c}$>0 and${g}_{m}\le 0$.

No research has yet been conducted to investigate the extended shopping time during the COVID-19 pandemic, featured with accounting for peace considerations. Building upon the Fig. 1, I am to develop a new shopping time function that incorporates the impact of both peace and COVID-19 simultaneously.

So Eq. (2) can be written as follows:

2 $$l_{tc}=[1-{n}_{t}-{n}^{ps}]$$

The shopping time function is represented by${n}^{{p}_{s}}$. The variable ${p}_{s}$, represents the level of peace. Any decrease in the peace variable or increase in the GPI index will result in an increase in shopping time. I have taken into consideration the impact of COVID-19 which influences the implementation of lockdown polices. This indicator is known as Case Fatality Risk (CFR), which is the proportion of individuals who die from a specific disease among all those diagnosed with the disease over a certain period of time [27]. Any increase in $CFR$ leads to stricter health lockdown policies, which in turn increases shopping time. I demonstrate this effect using the Eq. (3).

3 $${n}^{ps}={\text{g}}\left({c}_{t},{{\text{m}}}_{t}\right){e}^{{(-Pe+CFR)}_{t}}$$

Now, by replacing the Eq. (3) for the Eq. (2), the new leisure time is specified as follows:

4 $${l}_{tc}=1-n-{\text{g}}({c}_{t},{m}_{t})\begin{array}{cc}{l}_{tc}=1-{\text{n}}-{\text{g}}({c}_{t},{{\text{m}}}_{t})& {e}^{{(-Pe+CFR)}_{t}}\end{array}$$

By substituting Eq. (4) for leisure time in the utility function, we obtain the new utility function which includes COVID-19 pandemic and peace as follows:

5 $$\begin{array}{cc}{\text{u}}\left(c,m,l\right)\equiv [c,1-n-{\text{g}}({c}_{t},{m}_{t})& {e}^{{(-Pe+CFR)}_{t}}]\end{array}$$

Equation (6) represents the combined impact of both peace and COVID-19 in a shopping time model.

6 $${\text{g}}({c}_{t}, {{\text{m}}}_{t}) {e}^{(-Pe+CFR{)}_{t}}$$

According to Eq. (6), COVID-19 and peace both have a specific impact on the a shopping time and real balance. I can define a new definition of the precautionary motive rather than the definition of Keynes: "The desire for peace regarding the future cash equivalent of a certain proportion of total resources."

Now, I introduce a representative household's intertemporal utility function.

7 $$\begin{array}{cc}\sum_{j=0}^{\infty }{\beta }^{j}v[{c}_{t+j} , 1-{n}_{t+j}-\mathrm{ g}({c}_{t}, {{\text{m}}}_{t}) {e}^{(-{\varvec{P}}{\varvec{e}}+CFR{)}_{t}}]& 0\le \beta \le 1\end{array}$$

where $\beta$ is a discount factor that presents the time preference of a household, and ${c}_{t}$ is the per capita consumption at time t. The household's constraint is presented by Eq. (8).

*S*ubject to:

8 $${A}_{{\text{t}}}={\uptau }_{{\text{t}}}.{{\text{N}}}_{{\text{t}}}+\frac{\left(1+{{\text{i}}}_{{\text{t}}-1}^{{\text{g}}}\right){{\text{B}}}_{{\text{t}}-1}^{{\text{g}}}}{{{\text{P}}}_{{\text{t}}}}+\frac{\left(1+{{\text{i}}}_{{\text{t}}-1}^{{\text{m}}}\right){M}_{{\text{t}}-1}}{{{\text{P}}}_{{\text{t}}}}$$

${A}_{{\text{t}}}$ is a non-human wealth.${\uptau }_{{\text{t}}}$ is transfer payment. B is the total debt of a government. ${{\text{i}}}_{{\text{t}}-1}^{g}$ is the government bond yield and ${{\text{i}}}_{{\text{t}}-1}^{{\text{m}}}$ is the interest on money.${{\text{P}}}_{{\text{t}}}$ is the price index. ${M}_{{\text{t}}-1}$ is the stock of money.

9 $${Y}_{{\text{t}}}+\left(1-\updelta \right){{\text{K}}}_{{\text{t}}-1}+{A}_{{\text{t}}}\ge {C}_{{\text{t}}}+{{\text{K}}}_{{\text{t}}}+\frac{{{\text{M}}}_{{\text{t}}}}{{{\text{P}}}_{{\text{t}}}}+\frac{{{{\text{B}}}^{{\text{g}}}}_{{\text{t}}}}{{{\text{P}}}_{{\text{t}}}}$$

$${Y}_{{\text{t}}}$$ is the aggregate production function.$${Y}_{{\text{t}}}=F({{\text{K}}}_{{\text{t}}-1},{{\text{N}}}_{{\text{t}}},{\theta }_{t})$$, where $${{\text{K}}}_{{\text{t}}-1}$$ represents the aggregate stock of capital at the end of period t-1, $${{\text{N}}}_{{\text{t}}}$$ is the population, and $${\theta }_{t}$$ represents technology. $${\uptau }_{{\text{t}}}{{\text{N}}}_{{\text{t}}}$$ is the aggregate real value of any lump-sum transfers or taxes and, $$\updelta$$ is the rate of depreciation of physical capital. $${{\text{P}}}_{{\text{t}}}$$ =(1+$${\pi }_{t})$$ $${{\text{P}}}_{{\text{t}}-1}$$ and $${{\text{N}}}_{{\text{t}}}$$ =(1+$$n)$$ $${{\text{N}}}_{{\text{t}}-1}$$, where $${\pi }_{t}$$ and n are the inflation rate and net growth rate of the population.

Dividing both sides of the budget constraint (8) and (9) by the population ( ${{\text{N}}}_{{\text{t}}}$),

10 $${\frac{{A}_{{\text{t}}}}{{{\text{N}}}_{{\text{t}}}}=a}_{{\text{t}}}={\uptau }_{{\text{t}}}+\frac{\left(1+{{\text{i}}}_{{\text{t}}-1}^{g}\right){{{\text{B}}}^{{\text{G}}}}_{{\text{t}}-1}}{\left(1+{\pi }_{t}\right)\left(1+n\right){{\text{P}}}_{{\text{t}}-1}{{\text{N}}}_{{\text{t}}-1}}+\frac{\left(1+{{\text{i}}}_{{\text{t}}-1}^{{\text{m}}}\right){M}_{{\text{t}}-1}}{\left(1+{\pi }_{t}\right)\left(1+n\right){{\text{P}}}_{{\text{t}}-1}{{\text{N}}}_{{\text{t}}-1}}$$

or

11 $${a}_{{\text{t}}}={\uptau }_{{\text{t}}}+\frac{\left(1+{{\text{i}}}_{{\text{t}}-1}^{{\text{g}}}\right){{{\text{b}}}^{{\text{g}}}}_{{\text{t}}-1}}{\left(1+{\pi }_{t}\right)\left(1+n\right)}+\frac{\left(1+{{\text{i}}}_{{\text{t}}-1}^{{\text{m}}}\right){m}_{{\text{t}}-1}}{\left(1+{\pi }_{t}\right)\left(1+n\right)}$$

Now, the per capita budget constraint becomes:

12 $${y}_{{\text{t}}}+\frac{\left(1-\updelta \right)}{1+{\text{n}}}{{\text{k}}}_{{\text{t}}-1}+{a}_{{\text{t}}}\ge {c}_{{\text{t}}}+{{\text{k}}}_{{\text{t}}}+{{\text{m}}}_{{\text{t}}}++{{\text{b}}}_{{\text{t}}}^{{\text{g}}}$$

Now, ${Z(a}_{{\text{t}}},{{\text{k}}}_{{\text{t}}-1})$ is the value function.

13 $${Z(a}_{{\text{t}}},{{\text{k}}}_{{\text{t}}-1})=Max[{\text{V}}\left({c}_{t},1-{n}_{t}- \mathrm{ g}({c}_{t}, {{\text{m}}}_{t}) {e}^{(-{\varvec{P}}{\varvec{e}}+CFR{)}_{t}}\right)]+\mathrm{E}_\mathrm{t}{Z}\left(\alpha_{\mathrm{t}+1},\mathrm{k}_{\mathrm{t}}\right)=Max\left[\mathrm{v}\left(c_{t},{1}-n_t-g\left(c_t,{m}_t\right)e^{\left(-\varvec{Pe}+CFR\right)_t}\right)\right]+\upbeta {{\text{E}}}_{{\text{t}}}{\text{Z}}\left[{(\uptau }_{{\text{t}}+1}+\frac{\left(1+{{\text{i}}}_{{\text{t}}}^{{\text{g}}}\right){{{\text{b}}}^{{\text{g}}}}_{{\text{t}}}}{\left(1+{\pi }_{t+1}\right)\left(1+n\right)}+\frac{\left(1+{{\text{i}}}_{{\text{t}}}^{{\text{m}}}\right){m}_{{\text{t}}}}{\left(1+{\pi }_{t+1}\right)\left(1+n\right)}\right),({y}_{{\text{t}}}+\frac{\left(1-\updelta \right)}{1+{\text{n}}}{{\text{k}}}_{{\text{t}}-1}+{a}_{{\text{t}}}-{c}_{{\text{t}}}-{{\text{m}}}_{{\text{t}}}-{{{\text{b}}}^{{\text{g}}}}_{{\text{t}}})]$$

If I advance ${a}_{{\text{t}}}$ one period from Eq. (11), and replace the equivalent variables in Eq. (13) and also replace equivalent variables of the ${{\text{k}}}_{{\text{t}}}$ from Eq. (12), the necessary first-order conditions for labor, consumption, real money holdings, and real bond holdings should be derived as follows:

14 $$\frac{{\partial Z(a}_{{\text{t}}} ,{{\text{k}}}_{{\text{t}}-1}) }{\partial {n}_{{\text{t}}}}=\frac{\partial v}{\partial {l}_{t}}\frac{\partial {l}_{t}}{\partial {n}_{t}}+\upbeta {{\text{E}}}_{{\text{t}}}\frac{{\partial Z(a}_{{\text{t}}+1}{,{\text{k}}}_{{\text{t}}})}{\partial {k}_{t}}\frac{ \partial {k}_{t}}{\partial f}\frac{ \partial f}{\partial {n}_{t}}=0\underset{\Rightarrow }-{v}_{l}+\upbeta {{\text{E}}}_{{\text{t}}}\frac{{\partial Z(a}_{{\text{t}}+1},{{\text{k}}}_{{\text{t}}})}{\partial {k}_{t}}{f}_{n}=0$$

15 $$\frac{{\partial Z(a}_{{\text{t}}} ,{{\text{k}}}_{{\text{t}}-1}) }{\partial {c}_{{\text{t}}}}=\frac{\partial v}{\partial {c}_{t}}+\frac{\partial v}{\partial {l}_{t}}\frac{\partial {l}_{t}}{\partial g}\frac{\partial g}{\partial c}{e}^{(-{\varvec{P}}{\varvec{e}}+CFR{)}_{t}}-\upbeta {{\text{E}}}_{{\text{t}}}\frac{{\partial Z(a}_{{\text{t}}+1}{,{\text{k}}}_{{\text{t}}})}{\partial {k}_{t}}\frac{ \partial {k}_{t}}{\partial {c}_{t}}=0\underset{\Rightarrow }{v}_{c}+{v}_{l}{g}_{c} {e}^{(-Pe+CFR{)}_{t}}=\beta {E}_{t}\frac{{\partial Z(a}_{t+1},{k}_{t})}{\partial {k}_{t}}$$

16 $$\frac{{\partial Z(a}_{{\text{t}}},{{\text{k}}}_{{\text{t}}-1}) }{\partial {m}_{{\text{t}}}}=\frac{\partial v}{\partial {l}_{t}}\frac{\partial {l}_{t}}{\partial g}\frac{\partial g}{\partial {m}_{{\text{t}}}} {e}^{(-{\varvec{P}}{\varvec{e}}+CFR{)}_{t}}+\upbeta {{\text{E}}}_{{\text{t}}}\left[\frac{{\partial Z(a}_{{\text{t}}+1},{{\text{k}}}_{{\text{t}}})}{{\partial a}_{{\text{t}}+1}}.\frac{{\partial a}_{{\text{t}}+1}}{\partial {m}_{{\text{t}}}}+ \frac{{\partial Z(a}_{{\text{t}}+1},{{\text{k}}}_{{\text{t}}})}{\partial {k}_{t}}.\frac{\partial {k}_{t}}{\partial {m}_{{\text{t}}}} \right]=0\underset{\Rightarrow } -{v}_{l}{g}_{m} {e}^{(-{\varvec{P}}{\varvec{e}}+CFR{)}_{t}}+\upbeta {{\text{E}}}_{{\text{t}}}\left[\frac{{\partial Z(a}_{{\text{t}}+1},{{\text{k}}}_{{\text{t}}})}{{\partial a}_{{\text{t}}+1}}.\frac{\left(1+{{\text{i}}}_{{\text{t}}}^{{\text{m}}}\right)}{\left(1+{\pi }_{t+1}\right)\left(1+n\right)}- \frac{{\partial Z(a}_{{\text{t}}+1},{{\text{k}}}_{{\text{t}}})}{\partial {k}_{t}} \right]=0$$

17 $$\frac{{\partial Z(a}_{{\text{t}}},{{\text{k}}}_{{\text{t}}-1}) }{\partial {{{\text{b}}}^{{\text{g}}}}_{{\text{t}}}}=\upbeta {{\text{E}}}_{{\text{t}}}\left[\frac{{\partial Z(a}_{{\text{t}}+1},{{\text{k}}}_{{\text{t}}})}{{\partial a}_{{\text{t}}+1}}\frac{{\partial a}_{{\text{t}}+1}}{\partial {{{\text{b}}}^{{\text{g}}}}_{{\text{t}}}}+\frac{{\partial Z(a}_{{\text{t}}+1},{{\text{k}}}_{{\text{t}}})}{\partial {k}_{t}}\frac{\partial {k}_{t}}{\partial {{{\text{b}}}^{{\text{g}}}}_{{\text{t}}}}\right]=0 \underset{\Rightarrow }\upbeta {{\text{E}}}_{{\text{t}}}\left[\frac{{\partial Z(a}_{{\text{t}}+1},{{\text{k}}}_{{\text{t}}})}{{\partial a}_{{\text{t}}+1}}\frac{\left(1+{{\text{i}}}_{{\text{t}}}^{{\text{g}}}\right)}{\left(1+{\pi }_{t+1}\right)\left(1+n\right)}-\frac{{\partial Z(a}_{{\text{t}}+1},{{\text{k}}}_{{\text{t}}})}{\partial {k}_{t}}\right]=0$$

Removing $\frac{{\partial Z(a}_{{\text{t}}+1},{{\text{k}}}_{{\text{t}}})}{{\partial a}_{{\text{t}}+1}}$ in Eq. (16) by Eq. (17), then Eq. (18) is obtained:

18 $$\begin{array}{cc}-{v}_{l}{g}_{m}& {e}^{-({\varvec{P}}{\varvec{e}}-CFR{)}_{t}} =\frac{{{\text{i}}}_{{\text{t}}}^{g}-{{\text{i}}}_{{\text{t}}}^{{\text{m}}}}{1+{{\text{i}}}_{{\text{t}}}^{g}}\upbeta {{\text{E}}}_{{\text{t}}}\bigg[\frac{{\partial Z(a}_{{\text{t}}+1},{{\text{k}}}_{{\text{t}}})}{\partial {k}_{t}}\bigg]\end{array}$$

According to Eq. (14), $\upbeta {{\text{E}}}_{{\text{t}}}\frac{{\partial Z(a}_{{\text{t}}+1},{{\text{k}}}_{{\text{t}}})}{\partial {k}_{t}}=\frac{{v}_{l}}{{f}_{n}}$. By removing $\frac{{\partial Z(a}_{{\text{t}}+1},{{\text{k}}}_{{\text{t}}})}{\partial {k}_{t}}$ from Eq. (18),

replacing it with$\frac{{v}_{l}}{{f}_{n}}$, and omitting ${v}_{l}$ from both side of Eq. (18), finally, Eq. (19) is extracted.

19 $$-{g}_{m}{f}_{n} =\frac{{{\text{i}}}_{{\text{t}}}^{g}-{{\text{i}}}_{{\text{t}}}^{{\text{m}}}}{1+{{\text{i}}}_{{\text{t}}}^{g}}{e}^{({\varvec{P}}{\varvec{e}}-CFR{)}_{t}}$$

The left side represents the value of transaction time saved by holding additional money or the opportunity cost of holding money and can bee shown with ${V}_{m}$.

This indicates that as the level of peace increases, the value of transaction time saved by additional holding money increases and makes the household to hold less money. This means that a household living in a higher peaceful country holds less money than a one who lives in a less peaceful country. Unlike this, if CFR increases, then the value of transaction time saved by holding additional money will drop significantly. This persuades the household to hold more money. Therefore, households with higher CFR tend to hold more money compared to those in countries with lower CFR.

We know that in (19) ${g}_{m}<0$ then we define $-{g}_{m}{f}_{n}={V}_{m}$ $\ge 0$

According to the theory of loanable funds, discount rate affects both the interest on money and the bond yield. There is a positive relationship between the discount rate and the interest on money, but an inverse relationship between the discount rate and the price of government bonds and their yield [28], 3).

I simplify this relationship using Eq. (20):

20 $$\begin{array}{cc}\frac{{{\text{i}}}_{{\text{t}}}^{g}-{{\text{i}}}_{{\text{t}}}^{{\text{m}}}}{1+{{\text{i}}}_{{\text{t}}}^{g}}& =\frac{\varphi }{{R}_{t}}\end{array}$$

$\varphi$ is a parameter and R, is the discount rate. Now, I can rewrite Eq. (19):

${V}_{m}=\frac{\varphi }{{R}_{t}}$. ${e}^{({\varvec{P}}{\varvec{e}}-CFR{)}_{t}}$ And if I subtract "Ln" from both side:

21 $${{\text{ln}\ }(V}_{m})={\text{ln}}\left(\varphi \right)-{\text{ln}}\left({R}_{t}\right)+{Pe}_{t}-{CFR}_{t}$$

With differentiation from both sides and in a 3steady state, where only $({p}_{e}{)}_{t}$ varies:

22 $$\dot{V}=-\dot{R}+d(Pe)_t-d(CFR)_t=0\Rightarrow\dot{R}=d(Pe)_t-d(CFR)_t$$

Therefore, optimal monetary policy is a policy in which growth rate of discount rate ( $\dot{R}$) equals with the aggregation of changing in the level of paece and case fatality risk.

To measure the peace, Global Peace Index is used. We know that ${\varvec{d}}Pe=-d(GPI)$.Therefore, we rewrite Eq. (22) as follows:

23 $$\dot{R}=-d(GPI{)}_{t}-d(CFR{)}_{t}$$

According to Eq. 23, an increase in peace, (negative change in $I$,$d{(GPI)}_{t}<0$) would lead to an increase in the growth rate of discount rate. Conversely, a decrease in peace (positive change in $PI$,$d{(GPI)}_{t}>0$) would lead to a decrease in the growth rate of discount rate. Similarly, an increase in CFR would lead to a decrease in$\dot{R}$, while a decrease in CFR would lead to an increase in the$\dot{R}$. Therefore, we have:

24 $$\dot{R}=-[{\text{d}}(GPI{)}_{t}+d(CFR{)}_{t}]$$

This equation shows an optimal monetary policy condition or an optimal growth rate of discount rate with the inclusion of the new peace index during the COVID-19 pandemic. The article presents a monetary model that is innovative because no study has been found that focuses on changes in the CFR and peace index when determining the discount rate during biological and political impulses.

## Results

To calculate the optimal growth discount rate based on the Eq. (24), three groups of countries have been selected. Thanks to the Keynes' book "The Economic Consequences of the Peace," the rationale behind the selected countries is based on the economic and peace Indexes. In terms of the economy, the top 15 GDP countries in 2020 were selected as the first group, as shown in Table (1). These countries including the United States, China, Japan, Germany, United Kingdom, India, France, Italy, Canada, South Korea, Russia, Brazil, Australia, Spain, and Indonesia. They altogether account for 76% of the world's nominal GDP (https://globalpeoservices.com). Moreover, these countries represent approximately 55% of the world's population (https://www.worldometers.info/world-population).

**Table 1:** An optimal growth rate of discount rate for the top 15 GDP countries in2020

**Countries**	D(GPI)	Case	Dead	CFR%	Opt. Disc
USA	-0.036	20,642,688	365,187	1.77	-1.734
China	0.019	87,052	4634	5.32	-5.339
Japan	-0.01	230,304	3414	1.48	-1.47
Germany	-0.051	1,745,518	34,194	1.96	-1.909
UK	-0.036	2,483,039	73,609	2.96	-2.924
India	-0.005	10,286,329	150,036	1.46	-1.455
France	0.014	2,459,116	64,780	2.63	-2.644
Italy	-0.034	2,116,539	74,147	3.50	-3.466
Canada	-0.009	581,395	15,606	2.68	-2.671
South Korea	-0.032	60,740	900	1.48	-1.448
Russia	-0.04	3,159,297	57,019	1.80	-1.76
Brazil	0.052	7,675,973	194,976	2.54	-2.592
Australia	-0.01	28,405	909	3.20	-3.19
Spain	-0.022	1,971,003	50,837	2.58	-2.558
Indonesia	0.061	743,198	22,138	2.98	-3.041

Source**:** Calculated by Author

When it comes to peace, the 10 most peaceful countries, as shown in Table (2) are chosen as the second group. The third group includes the 10 least peaceful countries along with the Islamic Republic of Iran (IRI) which is displayed in Table (3). Although the Islamic Republic of Iran was not initially among the 10 least peaceful countries, it has been included and reviewed by international circles due to the Joint Comprehensive Plan of Action as a world peace treaty.

**Table 2:** An optimal growth rate of discount rate for the 10 most peaceful countries in 2020

**Countries**	D(GPI)	Case	Dead	CFR%	Opt. Disc
Iceland	0.014	5754	29	0.50	-0.514
Portugal	0	413,678	6906	1.67	-1.67
New Zealand	0.027	2162	25	1.16	-1.187
Austria	0.011	360,815	6222	1.72	-1.731
Denmark	-0.001	163,479	1298	0.79	-0.789
Singapore	-0.023	58,599	29	0.05	-0.027
Canada	-0.009	581,395	15,606	2.68	-2.671
Czech Republic	-0.007	718,983	11,848	1.65	-1.643
Switzerland	0.001	452,296	7882	1.74	-1.741
Slovenia	0.022	122,198	2697	2.21	-2.232

Source: Calculated by Author

**Table 3:** An optimal growth rate of discount rate for the 10 least peaceful countries plus Iran in 2020

**Countries**	D(GPI)	Case	Dead	CFR%	Opt. Disc
Afghanistan	1.58	52,513	2201	4.19	-5.77
Syria	-0.6	11,434	711	6.22	-5.62
Iraq	2.3	595,291	12,813	2.15	-4.45
South Sudan	0.22	3558	63	1.77	-1.99
Yemen	1.04	2099	610	29.06	-30.1
Democratic Republic of the Congo	-0.44	7107	108	1.52	-1.08
Somalia	1.34	4714	130	2.76	-4.1
Central African Republic	-1.14	4936	63	1.28	-0.14
Libya	-0.5	100,277	1478	1.47	-0.97
Russia	0.56	3,159,297	57,019	1.80	-2.36
IRI	2.84	1,225,143	55,223	4.51	-5.77

Source: Calculated by Author

When it comes to tables, the last column represents the optimal growth rate of the discount rate based on the Eq. (23), according to which the column dedicated to optimal discount rate presents the aggregation of column of D(GPI) and CFR. D(GPI) indicates the difference between the level of peace between 2019 and 42020. Cases and deaths for COVID-19 are extracted in December 2020 to 5calculate 6CFR.

These tables show that the monetary authority must simultaneously consider changes in CFR and GPI in order to adjust the optimal growth rate discount rate.

According to Table (2), the 10 most peaceful countries could increase the growth rate of discount rate since the absolute value of the d(GPI) is greater than the CFR.

According to Table (3), albeit the GPI decreased in Syria, Libya, the Democratic Republic of the Congo, and the Central African Republic, the growth rate of discount rate should be reduced because its absolute value is lower than the CFR. It is important to note that in some countries, such as the Iran, the monetary authority has not adjusted the discount rate based on the findings presented in Table (3).

## Discussion

Since the effect of COVID-19 on the GPI can be seen through the lockdown policies, which are considered as the proxy of CFR in this research in Fig. 1, the higher the CFR, the more severe the lockdown policy against COVID-19. Mathematically, the findings based on Eq. 23 show that the CFR should be added to the GPI for any optimal monetary decision making. The reason for this is that the tightening of lockdown policies and the increased need for drugs and medical equipment to deal with COVID-19 may raise the indicator "number and duration of internal and external conflicts" ultimately worsening the domain of "Society Safety and Security." Keeping people in quarantine can cause violent social behavior or the possibility of demonstrations against a lockdown policy. This can have an impact on the indicators of " Level of violent crime" and "Likelihood of violent demonstrations". Finally, the implementation of lockdown policies may require an increase in military spending which would affect the indicator of " Military expenditure as a percentage of GDP". Therefore, the new peace index should be adjusted by health indicators such as CFR.

It is crystal clear that all tables convey the message that not only the top 15 GDP countries of 2020 had to decrease the growth rate of discount rate but the 10 most peaceful countries as well as the 10 least peaceful countries plus Iran had to decrease it through the specific rule. The obtained results are comparable to an objective events in some countries. For example, in the USA, If the FOMC in the USA wanted to choose the discount rate in December of 2020 by Eq. 23, it should have used the $\frac{\left({r}_{2020}-{r}_{2019}\right)}{{r}_{2019}}$=-0.01734. ${r}_{2019}$ and ${r}_{2020}$ are the discount rates in December of 2019 and 2020 respectively.${r}_{2019}$ was 2.25 percent. Therefore, the discount rate would have been 2.21 percent in December 2020. However, according to the information from the Federal Reserve Economic Data,(FRED) website (https://fred.stlouisfed.org/series/INTDSRUSM193N), which presents all data on discount rates for many countries, the discount rate in the USA was actually 0.25 percent in the last month of 2020. But, what the FOMC did was reducing the growth rate by 88%, which was not optimal, contrary to the results of this research. This mistake by the FOMC was due to the fact that the decision regarding the discount rate did not take into account the global dimensions of peace and health. According to the International Monetary Fund in 2022, the USA experienced high inflation after COVID-19, [30].The consequence of this mistake can be observed in the inaccurate estimation of the discount rate due to the advent of COVID-19. This has resulted in the failure of the inflation targeting policy and an increase in core inflation within the US economy. Therefore, monetary authorities around the world such as the FOMC should account for the findings of this paper. The study proposes a new monetary rule based on the level of peace and health. Through so doing, the findings will helpt to manage economic instability by controlling excessive liquidity. During an epidemic like Covid-19, incorrect and excessive changes in monetary variables such as the discount rate can actually exacerbate economic instability and inflation. This can lead to social unrest, tensions, security issues, and accelerate the spread of infectious diseases worldwide. Instead, optimal changes in monetary variables can improve the health and peace conditions in a society by maintaining economic stabilit.

Excessivly lowering the discount rate during the time of COVID-19 can lead to increased debt and liquidity problem. This may further prolong the economy's return to a stable state. Therefore, to improve economic resilience of countries, case fatality risk should be considered to establish a new optimal criterion for monetary authorities to determine the appropriate discount rate. Such a criterion will help enhanceenhance economic resilience when dealing with a biological shock and enable the implantation of the United Nations' Sustainable Development Goals (SDGs) 16 (Peace, Justice, and Strong Institutions) and SDG 3 (Good Health and Well-Being) with minimal volatility.

To reach the goals set by the United Nations, it is recommended that the organization cooperates with the International Monetary Fund and the World Health Organization to create a document called the Optimal Currency Areas for Sustainable Peace and Health (OCASPAHA). This document will establish clear and coordinated monetary policy rules for countries grouped by their levels of Peace and CFR indexes. Countries that violate the protocols outlined in the document by implementing extreme monetary policies will be subject to fines through international legal channels. In addition, the author suggests making changes to the international monetary implementation rules related to bank management, such as Basel. In addition, the author suggests making changes to the international monetary implementation rules related to bank management, such as Basel. The author also recommends modifying monetary policy rules that assist monetary authorities in planning economies, such as Taylor [31] and Ball [32]. These adjustments and modifications should be based on peace and CFR indexes to prevent the excessive ease in financing due to on quantitative easing in the economy, and to provide a new opportunity for alternative non-inflationary and resistant alternative.

## Conclusions

By expanding Keynes' definition of the term "security" to include peace, the theoretical foundations of the relationship between uncertainty and the precautionary motive for holding money are updated and supplemented. The GPI can be used in monetary policies to measure economic risk and interest rates alongside the CFR during a biological shock such as COVID-19 pandemic. The results indicate that a decrease in peace and the outbreak of COVID-19 compelled monetary authorities to reduce the cost of holding money (discount rate) and decrease the cost of keeping money. This will increase global liquidity and may lead to inflation in coming years. Therefore,countries that contribute to a decline in global peace and health should be penalized by bearing the cost of imbalances caused by changes in the cost of holding money. This penalty increases the cost of holding money and opens up an opportunity for alternative financing. Additionally, the penalty should be based on a specific rule and logic outlined in the international peace treaty, as emphasized by Keynes at the conference of Versailles.

## Data Availability

Publicly available data are used in the study. All data relevant to the study are provided with the sources in the article.

## Footnotes

1 According to IEP, GPI, as an index for measuring the peace," Security is one of the domains of the Global Peace Index. Therefore, now, security is a part of the Peace Index.

2 Economic Policy Uncertainty

3 This condition is inspired by the study of Reis [29], who believes that in a steady-state, it is not necessary for money or interest rates to remain unchanged ([29], 132).

4 Data are used from https://www.visionofhumanity.org/wp-content/uploads/2020/10/GPI_2020_web.pdf

5 Data are used from the world bank in 2020 for case and dead of all tables.://www.worldometers.info/coronavirus

6 Since at the end of 2019, the recorded statistics were not from COVID-19, for most of the World, CFR in 2019 is considered zero, so CFR in 2020 also indicates changes in it. On the other hand, d(CFR) = CFR at the end of 2020.
